# Quality of life, socioeconomic profile, knowledge and attitude toward sexuality from the perspectives of individuals living with Human Immunodeficiency Virus^1^

**DOI:** 10.1590/0104-1169.3424.2542

**Published:** 2015-04-14

**Authors:** Meiry Fernanda Pinto Okuno, Gisele Cristina Gosuen, Cássia Regina Vancini Campanharo, Dayana Souza Fram, Ruth Ester Assayag Batista, Angélica Gonçalves Silva Belasco

**Affiliations:** 2 PhD, RN, Escola Paulista de Enfermagem, Universidade Federal de São Paulo, São Paulo, SP, Brazil; 3 MSc, Physician, Universidade Federal de São Paulo, São Paulo, SP, Brazil; 4 Doctoral student, Universidade Federal de São Paulo, São Paulo, SP, Brazil. RN, Escola Paulista de Enfermagem, Universidade Federal de São Paulo, São Paulo, SP, Brazil; 5 PhD, RN, Escola Paulista de Enfermagem, Universidade Federal de São Paulo, São Paulo, SP, Brazil; 6 PhD, Adjunct Professor, Escola Paulista de Enfermagem, Universidade Federal de São Paulo, São Paulo, SP, Brazil

**Keywords:** Quality of Life, Aged, Acquired Immunodeficiency Syndrome, Sexuality

## Abstract

**Objectives:**

to analyze the quality of life of "patients" with Human Immunodeficiency Virus and relate it to their socioeconomic profile, knowledge and attitudes toward sexuality.

**Method:**

crosssectional and analytical study with 201 individuals who are 50 years old or older. The Targeted Quality of Life and Aging Sexual Knowledge and Attitudes Scales were applied during interviews. Multiple Linear Regression was used in data analysis.

**Results:**

dimensions of quality of life more strongly compromised were disclosure worries (39.0), sexual function (45.9), and financial worries (55.6). Scores concerning knowledge and attitudes toward sexuality were 31.7 and 14.8, respectively. There was significant correlation between attitudes and the domains of overall function, health worries, medication worries, and HIV mastery.

**Conclusion:**

guidance concerning how the disease is transmitted, treated and how it progresses, in addition to providing social and psychological support, could minimize the negative effects of the disease on the quality of life of patients living with the Human Immunodeficiency Virus.

## Introduction

The Joint United Nations Programme on HIV/AIDS (UNAIDS) reports that more than 34 million people live with HIV worldwide: 30.7 million are adults and 3.4 million are younger than 15 years old, while 16.7 million are women. A total of 1.4 million are in Latin America, 350,000 of which live in Brazil, while estimates show that 250,000 Brazilians are infected but do not know that they are^(^ 1 ^)^.

In 2010, in Brazil, the incidence of the disease was higher among individuals aged between 40 and 49 years old, representing 24.8% of the cases, though an increase has been observed among individuals aged from five to 12 years old and those 50 years old or older^(^ 2 ^)^.

The growing number of 50 years old or older individuals living with HIV may be related to the fact they acquire the virus at an older age; they do not realize they may acquire the virus, probably because, as the disease came to notoriety, it was more associated with young individuals, intravenous drug users and homosexuals; and to the fact that medications improve sexual performance and favor the establishment of new and multiple sexual partnerships^(^ 3 ^)^. Additionally, some of the young population who became infected is now aging. Due to the efficacy of the antiretroviral therapy, there has been an increase in the number of individuals 50 years old or older with the disease^(^ 3 ^)^.

Even though sexuality is a basic need and an aspect of humankind that cannot be separated from other aspects of life, regardless of age, the aging process leads to some physical changes that sometimes affect one's ability to practice sex and have such pleasure with another person^(^ 4 ^)^. The knowledge of the patients addressed in this study concerning sexuality was associated with myths and changes in the sexual functioning of individuals as they age. 

Sexuality, as an important aspect of life, can lead to changes in the quality of life over time^(^ 5 ^)^. Arguably, information concerning sexuality contributes to a healthy and safe sexual life in old age^(^ 6 ^)^.

One's attitude toward sex is a result of social and sexual experiences^(^ 6 ^)^. Choosing a sexual partner, the frequency with which one practices sex, good sexual life, and interest in sex are positively associated with the health of adults and elderly individuals and may be reflected in a positive attitude toward sexuality and the prevention of diseases^(^ 7 ^)^.

A positive attitude among elderly individuals toward sexuality may favor an understanding that sexual activity is a natural process and that the sexual function remains throughout life^(^ 8 ^)^. The individuals interviewed in this study were assessed as to whether they had a positive attitude toward sex in old age or not; in Brazil, elderly individuals are those 60 years old or older^(^ 9 ^)^.

The presence of HIV/AIDS, as well as symptoms and complications associated with the disease, negatively affect the quality of life (QoL) of those living with the virus. Additionally, several sociodemographic, clinical, and psychosocial factors have been reported in the literature as variables that can impact the QoL of these individuals^ (10-11)^. In general, QoL dimensions include physical, psychological, social, interpersonal, environmental, and spiritual aspects that enable complementing morbidity and mortality models traditionally used to assess the impact of a given disease. In this context, assessing factors that interfere in the QoL of people living with HIV/AIDS is an important marker of disease and also important to implementing therapeutic interventions among this population^(^ 12 ^)^.

Identifying potentially modifiable factors of QoL is essential to making medical decisions, implementing health care measures and optimizing the use of resources in healthcare services to improve the well being of people living with HIV/AIDS^(^ 10 ^)^.

Since variables that affect the QoL of patients with HIV/AIDS have not yet been completely identified, this study's objectives were to analyze the quality of life of "patients" with HIV and relate it to their socioeconomic profiles, knowledge and attitudes toward sexuality.

## Method

This epidemiological, cross-sectional and analytical study was conducted in the outpatient clinic coordinated by the Infectious and Parasitic Diseases course administered at UNIFESP, between May 2011 and March 2012. A total of 201 female and male individuals with HIV/AIDS were included in the study, provided they were aged 50 years old or older, presented no cognitive deficit, were monitored by an infectious disease specialist, and confirmed their consent by signing free informed consent forms. Those who did not meet the inclusion criteria were excluded from the study.

We used a stratified random sampling method proportional to the average number of patients 50 years old or older, receiving care in the six months that preceded the study. The sample size computation took into account a degree of confidence equal to or greater than 80% and an alpha of 5%, based on characteristics such as age, gender, education, marital status, occupation, physical exercise, time since the disease was diagnosed, how the individual was infected, and comorbidities. The results showed a need to include 201 patients to achieve the desired objectives.

A structured questionnaire was used. It addressed information on age, gender, education, marital status, occupation, exercise, time since the disease was diagnosed, how the individual became infected, and comorbidities. Economic status was classified according to *Critério de Classificação Econômica Brasil* [Economic Criteria Classification], in which the final score, which represents an economic class (from A to E), is the sum of points concerning level of education and amount of consumer goods the individual has at home^(^ 13 ^)^.

The Targeted Quality of Life Instrument (HAT-QoL)^(^ 14 ^)^ was used. It is composed of 34 items addressing nine dimensions: overall function, life satisfaction, health worries, financial worries, medication worries, HIV mastery, disclosure worries, provider trust, and sexual function. The individual is instructed to consider his/her QoL in the last four weeks and answers are presented on a five-point Likert scale: "all the time", "most of the time", "some of the time", "a little of the time" and "never". The scores range from zero (worst situation) to 100 (best possible situation).

The Aging Sexual Knowledge and Attitudes Scale (ASKAS)^(15) ^was also used. It is composed of 20 questions addressing the construct "knowledge", ranging from 20 to 60, and eight questions addressing "attitudes," which range from 8 to 40. The options for answers to the knowledge questions include: true (one point); false (two points); and do not know (three points); the lower the score, the higher one's knowledge regarding sexuality in old age. The options for answering the questions addressing attitudes include: strongly disagree (one point), partially disagree (two points), do not agree or disagree (three points), partially agree (four points), and strongly agree (five points). The lower the score, the more favorable is one's attitude toward sexuality in old age.

The patients were invited to participate in the study when they visited the clinic for routine exams or medical appointments. If they consented, they were individually interviewed in a private outpatient clinic. The researcher read the instruments' items only once and the interviews lasted 40 minutes, on average. 

Descriptive statistics was used to present the participants' sociodemographic, economic, and clinical-epidemiological characterizations, comorbidities, exercise patterns, and the results of the ASKAS. Average, standard deviation, median, minimum and maximum were computed for the continuous variables, while frequencies and percentages were computed for categorical variables.

A Linear Regression model was used to verify which independent variables (sociodemographic, economic, clinical-epidemiological, comorbidities, and exercise) better related with each domain of the HAT-QoL and the ASKAS. Multiple Linear Regression (stepwise regression) was then performed to select the set of variables that better explained the dependent variables (HAT-QoL and *ASKAS*). Analyses were performed using Statistical Package for the Social Sciences, version 1.9, and a level of significance of p<0.05 was used.

This study was approved by the Institutional Review Board at the Federal University of São Paulo (UNIFESP) No. 0182/11.

## Results

The patients' ages ranged from 50 to 74 years old; the ratio was 1.75 men for each woman; 67.7% did not have a stable partner; 9.5% had no income; only 14.4% had a bachelor's degree; and most (61.7%) individuals belonged to economic classes C, D, or E. Time since diagnosis as disclosed ranged from six months to 30 years, and heart diseases were the most frequent comorbidities (34.3%), as shown in Table 1.

**Table 1 -:** Sociodemographic, economic and morbidity characteristics of patients living with HIV/AIDS. São Paulo, SP, Brazil, 2012

Characteristics		n = 201	%
Age (years)^*^		56 (50 - 74)	
Gender^†^			
	Male	128	63.7
	Female	73	36.3
Race^†^			
	Caucasian	136	67.7
	Afro-descendant	27	13.4
	Mixed	38	18.9
Marital status^†^			
	Single/Divorced	103	51.3
	Married	65	32.3
	Widowed	33	16.4
Occupation^†^			
	Retired/Pensioner	106	52.7
	Employed	76	37.8
	Unemployed	10	5.0
	Homemaker	9	4.5
Education^†^			
	Illiterate/Incomplete elementary school	64	31.8
	Middle school	44	22.0
	High school	64	31.8
	Bachelor’s degree	29	14.4
Individual monthly income (Brazilian Real)^*^		1200 (0 - 9000)	
Economic Class^†^			
	A + B	77	38.3
	C + D + E	124	61.7
Time since the infection was diagnosed (years)^*^		12 (0.5 - 30.0)	
Comorbidities^†^			
	None	59	29.4
	Heart diseases^ ‡^	69	34.3
	Retinopathies	49	24.4
	Neoplasia	24	11.9

* Median (maximum-minimum)

† Absolute frequency and percentage

‡ Heart diseases: hypertension, peripheral vascular disease, and arterial, and coronary artery disease.

Table 2 shows that the most compromised domains of the QoL scale were disclosure worries (39.0), sexual function (45.9), and financial worries (55.6). The average scores of the ASKAS' knowledge and attitudes domains were 3.7 and 14.8, respectively, showing that this population has knowledge and attitudes favorable to sexuality.

**Table 2 -:** Average values of the HAT-QoL's and ASKAS' domains among patients living with HIV/AIDS. São Paulo, SP, Brazil, 2012

Domains		Average (± standard deviation)
HAT-QoL (n= 201)		
	Overall function	79.39 (20.9)
	Life satisfaction	71.9 (24.4)
	Health worries	83.2 (22.9)
	Financial worries	55.6 (37.5)
	Medication worries	88.7 (17.3)
	HIV mastery	77.8 (33.6)
	Disclosure worries	39.0 (27.3)
	Provider trust	72.2 (30.7)
	Sexual function	45.9 (43.5)
ASKAS		
	Knowledge	31.7 (6.7)
	Attitude	14.8 (6.8)

Table 3 shows that some sociodemographic, economic and clinical characteristics of elderly individuals with HIV/AIDS favored diverse QoL domains. Exercising, having knowledge of the disease for a longer period, belonging to economic class A or B, having a higher level of education, being unemployed, being aware of how one acquired the disease, being a man, Caucasian, and younger than 60 years old were significant aspects to having higher QoL scores. 

**Table 3 -:** Variables associated with the HAT-QoL's domains in the multiple linear regression analysis. São Paulo, SP, Brazil, 2012

HAT-QoL	Variables	Coefficient	p value	R^2*^
Overall function	Exercise (yes x no)	9.28	0.0018	0.0489
Life satisfaction	Exercise (yes x no)	7.85	0.0239	0.0465
	Longer time since diagnosis (years)	0.58	0.0449	
Health worries	Longer time since diagnosis (years)	0.81	0.0033	0.0436
Financial worries	Economic class (A and B x C, D and E)	13.38	0.0147	0.0303
HIV mastery	Education x illiterate	14.05	0.0061	0.0381
Disclosure worries	Employed x Unemployed	-13.03	0.0013	0.0627
	Age (55-59 years old x ≥60 years old)	8.39	0.0425	
Provider trust	How acquired the infection (know x do not know)	11.41	0.0262	0.0498
	Education x illiterate	-9.85	0.0321	
Sexual function	Male x female	25.00	0.0001	0.1627
	Age (55-59 years old x ≥60 years old)	16.99	0.0065	
	Race (Caucasian x non-Caucasian)	-15.79	0.0127	
	Economic class (AB x CDE)	12.98	0.0349	

* Coefficient of determination. Stepwise was the method of selection used.

No variable was significantly associated with the medication worries domain.

Table 4 shows that female and unemployed patients were those with greater knowledge concerning sexuality in old age, while those with higher levels of education and who were younger than 60 years old were those who presented more favorable attitudes toward sexuality among elderly individuals (attitudes domain).

**Table 4 -:** Sociodemographic variables associated with the ASKAS' domains in the multiple linear regression analysis. São Paulo, SP, Brazil, 2012

ASKAS	Variables	Coefficient	p value	R^2^*
Knowledge	Male x Female	-4.81	<0.0001	0.1436
	Employed x Unemployed	-1.96	0.0359	
Attitudes	Education x illiterate	-2.82	0.0066	0.0638
	Age (55-59 years old x ≥60 years old)	-2.15	0.0374	

* Coefficient of determination. Stepwise was the method of selection used.

There were significant associations between the ASKAS' attitudes domain and the HAT-QoL's overall function, health worries, medication worries, and HIV mastery. The ASKAS' knowledge domain, however, was not significantly associated with any HAT-QoL domains, as shown in Table 5.

**Table 5 -:** Multiple linear regression analysis between the ASKAS' attitude domain and the HAT-QoL's domains. São Paulo, SP, Brazil, 2012

HAT-QoL	p value	R^2^*
Overall function	0.0363	0.0218
Health worries	0.0010	0.0527
Medication worries	0.0091	0.0346
HIV mastery	0.0017	0.0483

* Coefficient of determination. Stepwise was the method of selection used.

## Discussion

Some characteristics of this study's population such as age, being mostly men, a low level of education, and low purchasing power are similar to the results found in another study conducted in Porto Alegre, RS, Brazil with HIV seropositive patients 50 years old or older, though most were employed and 41.9% were either married or lived with a partner^(^ 16 ^)^.

The scores of the HAT-QoL's domains were as follows: disclosure worries (39.03); sexual function (45.96); and financial worries (55.64). These scores were also similar to those found in studies conducted in Porto Alegre and a city in the interior of São Paulo, showing that regardless of the place of residence, the aspects of QoL most compromised are common among patients^(^ 11 ^,^ 16 ^)^.

Disclosure worries, the domain with the lowest score, may reflect the stigma and discrimination experienced by individuals with HIV/AIDS, which constantly generates a negative impact on people's QoL. In the absence of any intervention to fight stigmatization, these individuals will probably report dissatisfaction with life, evidenced by a decreased level of pleasure with life and social life^(^ 17 ^)^.

The compromised sexual function among this study's patients may be explained, in part, by the routine use of condoms, fear of rejection, of HIV superinfection, that the virus gets stronger, and fear of transmitting the virus, lack of trust in the partner, lack of sexual desire, and not considering sex to be an important part of life. Many participants of another study reported that HIV affected their sexual function^(^ 18 ^)^.

Lower QoL portrayed in the financial worries domain is probably related to the low income of individuals with the disease, which makes survival more difficult. QoL is closely linked to socioeconomic matters and social inclusion^(^ 10 ^)^.

This study's interviewees presented average scores of 31.7 on the ASKAS concerning knowledge of sexuality among elderly individuals, which ranged from 20 to 60, i.e., 29.4% of the total score. They scored 14.8 on the scale concerning attitudes toward sexuality in old age, which ranges from 8 to 40, i.e., 21.4% of the total score. An American study conducted with gynecologists showed average scores of 49.0 for the ASKAS' knowledge domain. Scores for this domain range from 35 to 105, and the score obtained is equivalent to 20.0% of the total score. Participants also scored 81.0 in the attitude domain, the scores of which range from 26 to 182, i.e., they scored 35.2% of the total score. This result shows that patients with HIV addressed in this study presented lower levels of knowledge and more favorable attitudes toward the sexuality of elderly individuals when compared to American physicians^(^ 19 ^)^.

The favorable attitudes toward sexuality in old age found among these individuals suggest they are sexually active; however, better knowledge of sexuality does not mean less risk of infection and educational practices are required to prevent the spread of the disease^(^ 7 ^)^.

The multiple linear regression analysis performed between the HAT-QoL's domains and other variables shows that exercise is associated with higher scores for overall function and life satisfaction. The relationship between exercise and overall health has been positively reported in recent decades among individuals with the virus. Physical exercise may positively influence immunological factors, increasing the production of natural antibodies, which in turn, can delay the progression of HIV/AIDS^(^ 20 ^)^.

Life satisfaction and health worries were domains with higher average scores that were associated with a longer period since HIV was diagnosed. One study conducted with seropositive individuals aged 50 years old and older reports that most react negatively when they received the diagnosis, though aging with HIV implied self-acceptance, wisdom, and a positive attitude toward life, essential aspects to maintaining life satisfaction and QoL^(^ 18 ^)^.

Patients belonging to economic classes A and B scored higher in the financial worries domain, showing that financial support is a positive aspect for QoL. The HIV epidemic is associated with a higher number of women and young individuals, internalization, aging and impoverishment^(^ 3 ^)^. Social exclusion triggered by being HIV positive leads individuals to experience social vulnerability. People with the disease who have a low level of education and low purchasing power have limited access to health, education, housing and food^(^ 17 ^)^. A low level of education also influences one's occupational options. Low income and poor socioeconomic condition influence the access of people living with HIV/AIDS to preventive measures and integral healthcare^(^ 11 ^)^.

The HIV mastery domain presented significantly higher scores among those with higher levels of education. Another study reports that lower education levels among infected people 50 years old or older hinders access to essential AIDS-related information, such as knowledge regarding antiretroviral medications that can control the disease. This information indicates that a low level of education may interfere in adherence to antiretroviral medication, as it influences one's understanding of the importance of using medications and accessing the treatment^(^ 21 ^)^.

Even though the average score obtained in the disclosure worries domain was low, it was better among unemployed individuals and those younger than 60 years old. Working individuals obtained the worst scores, possibly due to fear of discrimination and the possibility of losing their jobs. Many employers do not hire seropositive individuals due to prejudice, to the side effects caused by antiretroviral medications that may interfere in their productivity and to patients' needs to miss work days for consultations and exams^(^ 17 ^)^.

Social moral overload that patients experience seems to worsen with age. Another study conducted with elderly individuals with HIV/AIDS identified that the diagnosis impacted the individuals' affection-bonds, family ties and friendships. Fear of rejection was a major factor influencing the decision whether or not to disclose the diagnosis to the individuals' social circles^(^ 4 ^)^.

Being aware of the forms of transmission and a higher level of education were associated with higher scores on the HAT-QoL's provider trust domain. Higher levels of education may favor understanding about the disease and medication therapy, which is relevant for treatment adherence. Another study reports that individuals who do not adhere to the use of antiretroviral medication had lower levels of education than those who adhere to the treatment^(^ 19 ^)^. The lower scores found regarding the provider trust domain, may be explained, in part, by the fear some patients hold of being judged by healthcare providers and associated with homosexuality, drug users, and sex workers^(^ 22 ^)^. The sexual function domain showed that being a Caucasian man belonging to economic classes A or B was associated with higher scores. The maintenance of affective-sexual relationships is essential in the lives of individuals with HIV/AIDS as a contribution to better QoL^(^ 11 ^)^. Higher levels of education may be associated with the maintenance of sexual desire in the later stages of adult life^(^ 23 ^)^.

The study showed that some sociodemographic and clinical characteristics of patients with HIV/AIDS favored diverse domains of QoL. Physical exercise, being aware of the diagnosis for longer periods of time, belonging to economic classes A or B, having higher levels of education, being unemployed, and being aware of how the disease was acquired, being male and Caucasian, and younger than 60 years old were significant aspects to maintaining higher QoL scores.

The results of another study conducted with HIV/AIDS patients corroborate this study's findings and indicate variables that were positively associated with the domains of QoL: higher education and income^(^ 11 ^)^.

According to the ASKAS, women and unemployed individuals addressed in this study presented greater knowledge concerning sexuality in old age. Women in late stages of adult life remain interested in sexual life, feeling physically and mentally well to have sex, and in finding information and help concerning sexuality on the internet^(^ 24 ^)^.

Unemployed people perhaps presented greater knowledge regarding sexuality due to having more time to seek public health services. Employed individuals have little time to seek these services because their hours of work and those of the health services are not always reconcilable for working individuals^(^ 25 ^)^.

The interviewees who presented a better attitude toward sexuality were those with higher levels of education and who were younger than 60 years old. Another study reports that individuals with higher educational levels assigned greater importance to sex in their relationships with their spouses, showing that education seems to play an important role in people's attitudes toward sexuality^(^ 23 ^)^.

Sexuality in old age is a subject neglected by society, healthcare providers, and elderly individuals themselves as if love and even sex are no longer within the array of interests of those of advanced age^(^ 5 ^)^. It may be one of the reasons patients older than 60 years of age presented a less favorable attitude toward sexuality in old age.

More favorable attitudes toward sexuality were associated with the domains of overall function, health worries, medication worries, HIV mastery and addressed in the HAT-QoL in the multiple linear regression analysis. No studies related the HAT-QoL and the ASKAS, which hindered the comparison of results.

## Conclusion

The variables of physical exercise, long period since diagnosis, better economic conditions, higher level of education, being unemployed, being younger than 60 years old, being aware how transmission occurred, being a man, and being Caucasian were associated with one or more of the HAT-QoL's domains and increased its scores. Women and unemployed individuals had greater knowledge concerning the sexuality of elderly individuals, while patients with higher education levels and who were younger than 60 years of age showed more favorable attitudes toward sexuality in old age. Significant association was found between the HIV-QoL's domains overall function, health worries, medication worries, and HIV mastery with the ASKAS' attitude toward sexuality in old age domain.

The implementation of effective measures to prevent diseases, protect and maintain the QoL of people who age while having HIV/AIDS is necessary and urgent. Assessment and nursing interventions such as guidance concerning routes of transmission, treatment and how the pathology progresses, in addition to social and psychological support, could minimize the negative effects of the disease on the QoL of people living with HIV/AIDS.

This study's limitations include the fact that the participants were selected from a limited area of São Paulo and most were men. Men and women may have different experiences with HIV, which would impact QoL differently. This study's results cannot be generalized because they refer to specific characteristics of a given Brazilian region, although these results do allow building a perspective on QoL and its relationship with socioeconomic aspects, knowledge and attitudes toward sexuality in old age of individuals with HIV/AIDS. It can also provide useful information to support health policies concerning prevention and treatment.

Nursing assessments and interventions such as guidance regarding forms of transmission, treatment of and the progression of HIV/AIDS, in addition to social and psychological support, may minimize negative effects of the disease on the QoL of HIV-positive individuals.
